# Neuroprotective Properties of Cardoon Leaves Extracts against Neurodevelopmental Deficits in an In Vitro Model of Rett Syndrome Depend on the Extraction Method and Harvest Time

**DOI:** 10.3390/molecules27248772

**Published:** 2022-12-10

**Authors:** Mariachiara Spennato, Ottavia Maria Roggero, Simona Varriale, Fioretta Asaro, Angelo Cortesi, Jan Kašpar, Enrico Tongiorgi, Cinzia Pezzella, Lucia Gardossi

**Affiliations:** 1 Department of Chemical and Pharmaceutical Sciences, University of Trieste, Via L. Giorgieri 1, 34127 Trieste, Italy; 2 Department of Life Sciences, University of Trieste, Via L. Giorgieri 5, 34127 Trieste, Italy; 3 Department of Chemical Sciences, University Federico II of Naples, Via Cinthia, 4, 80126 Napoli, Italy; 4 Department of Engineering and Architecture, University of Trieste, Via Alfonso Valerio 6/A, 34127 Trieste, Italy

**Keywords:** cardoon leaves, plant extracts, bioactive molecules, Rett syndrome, bioeconomy, supercritical carbon dioxide, Naviglio^®^

## Abstract

This study investigates the bioactive properties of different extracts of cardoon leaves in rescuing neuronal development arrest in an in vitro model of Rett syndrome (RTT). Samples were obtained from plants harvested at different maturity stages and extracted with two different methodologies, namely Naviglio^®^ and supercritical carbon dioxide (scCO_2_). While scCO_2_ extracts more hydrophobic fractions, the Naviglio^®^ method extracts phenolic compounds and less hydrophobic components. Only the scCO_2_ cardoon leaves extract obtained from plants harvested in spring induced a significant rescue of neuronal atrophy in RTT neurons, while the scCO_2_ extract from the autumn harvest stimulated dendrite outgrowth in Wild-Type (WT) neurons. The scCO_2_ extracts were the richest in squalene, 3ß-taraxerol and lupeol, with concentrations in autumn harvest doubling those in spring harvest. The Naviglio^®^ extract was rich in cynaropicrin and exerted a toxic effect at 20 µM on both WT and RTT neurons. When cynaropicrin, squalene, lupeol and 3ß-taraxerol were tested individually, no positive effect was observed, whereas a significant neurotoxicity of cynaropicrin and lupeol was evident. In conclusion, cardoon leaves extracts with high content of hydrophobic bioactive molecules and low cynaropicrin and lupeol concentrations have pharmacological potential to stimulate neuronal development in RTT and WT neurons in vitro.

## 1. Introduction

Nutraceuticals or functional foods with antioxidant properties have recently been the object of intensive investigation due to their capacity to act on the triad of conserved core mechanisms underlying brain damage, which include oxidative stress, neurotrophic factors deficiency and inflammation [1,2]. Most plant extracts and pure bioactive ingredients typically show a polypharmacological profile [3,4] but the molecular mechanisms underlying their biological activity and the synergic action among the compounds of a phytochemical pool are mostly unknown. More importantly, natural extracts consist of complex mixtures whose composition depends upon the extraction methods employed [5]. Green tea polyphenols, resveratrol (a non-flavonoid polyphenol of red grapes), and curcumin (a polyphenol present in turmeric, *Curcuma longa*) are examples of such natural products. More recently, Omega 3 supplements have also been considered for neurodevelopmental brain diseases [6]. Interestingly, Silymarin, which is a mixture of polyphenolic flavonolignan, isolated from the seeds of *Silybum marianum*, has recently been introduced as supportive treatment for Alzheimer’s and Parkinson’s neurodegenerative diseases [7]. 

Cardoon (*Cynara cardunculus* L.), from the Asteraceae family, is a wild robust perennial species native to the Mediterranean basin [8] and has been cultivated in this region since ancient times with high productivity. It is naturally present in harsh habitat conditions characterized by high temperature, salinity [9] and drought [10,11]; therefore, it represents a valuable model of biomass for biorefinery development not competing for land dedicated to food crops. Cardoon biomass already finds applications in pulp and paper production and power generation, but it can also be valorized for the production of bioplastics, bio-lubricants [12], biopesticides, and ingredients for the cosmetic sector. More recently, cardoon biomass was used as sustainable feedstock for the production of polyhydroxyalkanoates (PHAs) [13]. Finally, cardoon biomass is also a rich source of valuable phytoconstituents, such as polyphenols and terpenoids, with well-known nutraceutical and pharmaceutical properties [14]. The leaves are known for their therapeutic potential as a diuretic, choleretic, cardiotonic, antidiabetic and anti-hemorrhoidal agent [5] as well as anti-inflammatory, anticancer, antioxidant, hepatoprotective, hypolipidemic, and antidiabetic activity [14]. 

Recent studies have shown the antioxidant activity of leaves extracts is strictly related to the polyphenol fraction [15,16]. In this respect, several extraction methods of bioactive molecules from cardoon leaves were previously described. Generally, leaves (dry or fresh) are mixed with different polar solvents, such as ethanol, methanol, acetone, or alcoholic solutions, and incubated with shaking [17,18,19]. An alternative to these traditional extraction methods was the use of the Naviglio^®^ extractor, based on a solid-liquid dynamic extraction, which allows the recovery of phenolic compounds from different types of solid matrixes, at short extraction times, with high yields and easiness of scalability [20]. 

This study explores cardoon biomass as a source of bioactive ingredients for application in the treatment of Rett syndrome (RTT), a paradigmatic neurodevelopmental disorder that presents the classical triad of brain disease core mechanisms [21]. Even if RTT is a rare genetic disease affecting 1:10.000 girls worldwide, it represents the second leading genetic cause of mental retardation in females [21]. RTT is mainly caused by mutations in the X-linked MeCP2 gene [21] and is characterized by delayed neuronal development, leading to brain atrophy with smaller neurons having decreased complexity of neuronal dendritic processes [22]. Clinically, RTT patients show developmental arrest, loss of speech and motor abilities, seizures, breathing abnormalities, and behavioral problems including autism [23,24,25]. More specifically, using an in vitro model of RTT based on primary neuronal hippocampal cultures from MeCP2 gene-deleted mice (MeCP2^-/y^ mice) [26,27], we have analyzed the effect of different extracts of cardoon leaves obtained from plants harvested at different maturity stages and extracted with two different methodologies, namely the Naviglio^®^ and the supercritical carbon dioxide (scCO_2_) extraction methods. The characterization of the main components of the different extracts allowed for the correlation of the observed beneficial or toxic biological responses to different composition profiles of the extracts.

## 2. Results and Discussions

### 2.1. Characterization of Cardoon Leaves Extracts

While scCO_2_ is specific for the extraction of very hydrophobic fractions, the Naviglio^®^ method is effective in extracting phenolic compounds and less hydrophobic components thanks to the action of polar solvents. More generally, supercritical fluids extraction (SFE) has the main advantage of process flexibility and allows for the elimination of polluting organic solvents, thereby avoiding expensive post-processing of the extracts for solvent elimination [28]. 

**Figure 1:**
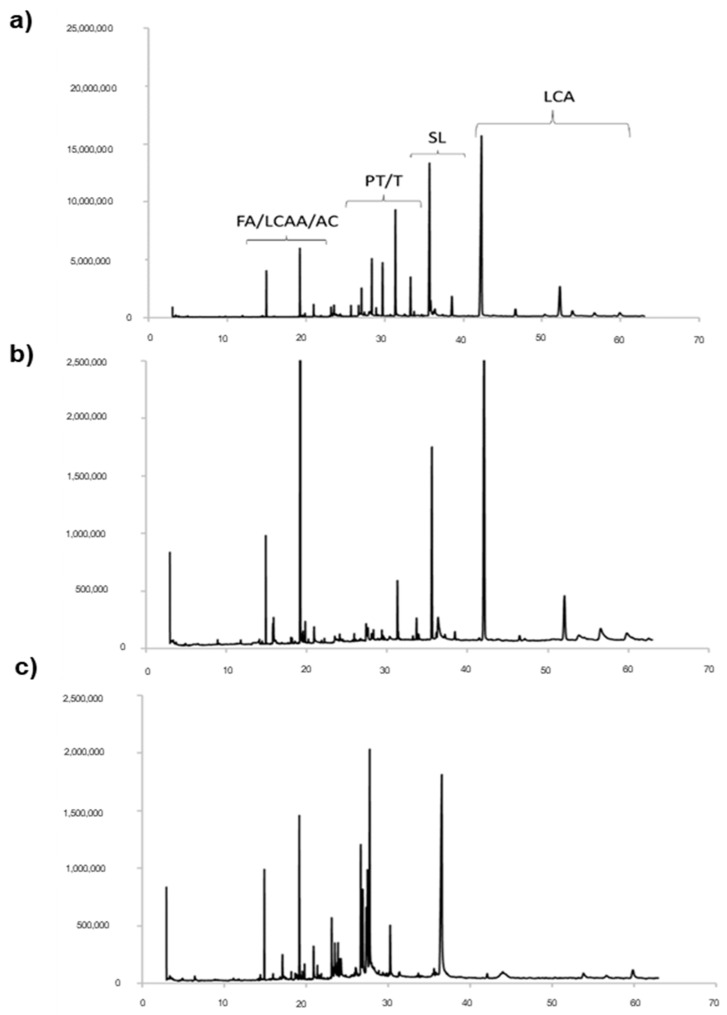
GC-MS chromatograms of the three extracts from cardoon leaves obtained using different extractive technologies: (**a**) autumn harvest with supercritical CO_2_ (scCO_2_Au); (**b**) spring harvest with supercritical CO_2_ (scCO_2_Sp); (**c**) spring harvest using Naviglio^®^ technology (NaviglioSp). FA = fatty acids; LCAA = long-chain aliphatic alcohols; AC = aromatic compounds; PT = pentacyclic triterpenes; T = triterpenes; SL= sesquiterpene lactone; LCA = long-chain alkanes.

The obtained samples of cardoon leaves extracts (CLEs) are labelled in the following text as scCO_2_Au, scCO_2_Sp and NaviglioSp, where Au and Sp refer to leaves collected in autumn and spring, respectively, and scCO_2_ and Naviglio indicate the supercritical CO_2_ extraction and Naviglio^®^ extraction technologies, respectively. The different CLEs were characterized by Gas Chromatography-Mass Spectrometry (GC-MS) and Nuclear Magnetic Resonance (^1^H NMR) spectroscopy. Figure 1 reports a comparison of the GC-MS chromatograms of the extracts obtained using supercritical CO_2_ from cardoon leaves collected in spring (scCO_2_Sp) and autumn (scCO_2_Au) (Figure 1a,b). Moreover, the leaves collected in spring were also extracted by means of Naviglio^®^ methods (NaviglioSp). 

The GC-MS analysis provides qualitative and quantitative information on the chemical components, which were identified by comparing the corresponding mass values with data available from the NIST14s database and the literature [29]. The predominant chemical species are pentacyclic triterpenes and sesquiterpenes, volatile lipophilic molecules known for their antioxidant activity [5,30]. Cynaropicrin and grosheimin (RT = 35.61–37.25 min) are sesquiterpene lactones known for being present in the highest amount in cardoon leaves as compared to other parts of the plant [5,31,32]. The GC-MS chromatograms (Figure 1) also indicate the presence of fatty acids (RT = 19.18 min), in particular linolenic acid [33], long-chain aliphatic alcohols (RT = from 19.50 to 22 min) and traces of aromatic compounds. Previous studies already indicated that fatty acids, especially saturated ones, are mainly concentrated in the leaves [5]. The signal at RT = 31.26 min is ascribable to squalene, whereas the signals corresponding to pentacyclic triterpenes fall in the range 23.45–29.73 min. Finally, the signals of hydrophobic long-chain alkanes are visible in the range 40–42.5 min. 

**Figure 2:**
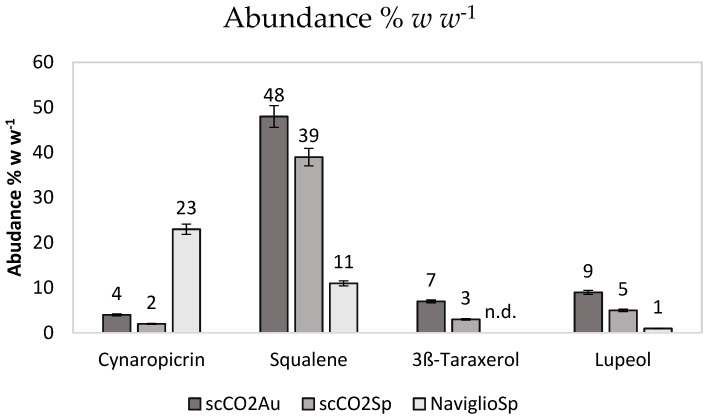
Abundance % *w w*^−1^ of each bioactive molecule measured by GC-MS. Data are the results of triplicated analysis and standard deviations are reported.

We then focused our attention on the four bioactive molecules reported in Table 1, which are abundant in the extracts, although with quantitative differences that are ascribable to the extractive methods (Figure 2). They are three triterpenes (squalene, taraxerol and lupeol) and a sesquiterpene (cynaropicrin) known for their bioactivities, which are relevant for the purpose of this investigation. The leaves extracts were analyzed by GC-MS to evaluate the concentration of the four biomolecules (Table 1). 

Squalene is a triterpene that is an intermediate in cholesterol biosynthesis pathways, widely distributed in nature [34]. Experimental studies have shown that squalene can effectively inhibit chemically induced skin, colon, and lung tumorigenesis in rodents [35]. Taraxerol is a triterpene that has been isolated from several plant species, and its various pharmacological properties have already been identified, such as the acetylcholinesterase (AChE) inhibition activity in vitro. Taraxerol has anti-amnesic activity that may hold significant therapeutic value in alleviating certain memory impairments observed in Alzheimer disease [36]. Lupeol is a significant lupene-type triterpene isolated in plants, fungi, and the whole animal kingdom. This bioactive molecule has several biological effects: anticancer, antiprotozoal, chemo-preventive, and anti-inflammatory properties [37]. Cynaropicrin is a sesquiterpene lactone that was isolated from artichoke (*Cynara scolymus* L.) in 1960 for the first time and was also found later in *Cynara cardunculus* L. Cynaropicrin has important pharmacological activities, such as antitumoral, anti-inflammatory, anti-trypanosome and anti-hepatitis C virus properties, among many others [38].

Figure 2 shows the abundance in % *w w*^−1^ of each bioactive molecule (% *w w*^−1^ i.e., the weight of the molecule of interest with respect to the total extract weight used for GC-MS analysis). 

The extracts obtained using supercritical CO_2_, specific for very hydrophobic molecules, are very rich in squalene. The rest of the hydrophobic extracts is mainly composed by waxes and fatty acids. Notably, there is an increased amount of squalene in the autumn extract as compared to leaves collected in spring. The spring extract obtained with the Naviglio method, employing ethanol as solvent, is the richest in cynaropicrin. The harvesting and maturity stages are crucial in determining the chemical composition of natural products obtained from plant tissues, with early maturity coinciding with the highest content of phenolic compounds, as already reported for various plant species [39].

### 2.2. Characterization of Cardoon Leaves Extracts Using NMR Spectroscopy

**Figure 3:**
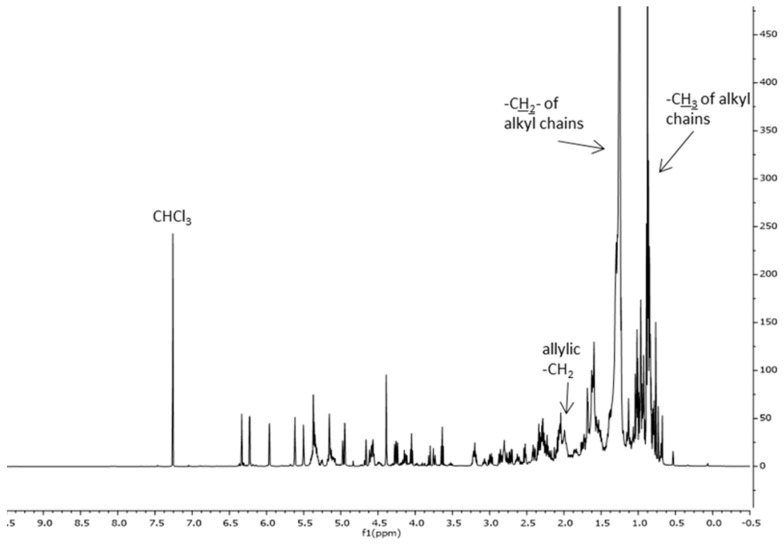
^1^H NMR spectrum of the CLE scCO_2_Sp. ^1^H NMR (500 MHz, CDCl_3_): δ: 0.54, –CH_3_ of sterols; δ: 0.69, –CH_2_ of triterpenes; δ: 0.85–0.93, –CH_3_ of alkyl chains; δ: 1.20–1.42, –CH_2_ of alkyl chains; δ: 1.6, –CH_2_CH_2_COOH of fatty acids; δ: 2.06, –CH_2_-CH=CH-; δ: 2.23 –CH_2_COO; δ: 3.5–6.5 alkene signals–compare also Figure 4.

Figure 3 reports, as an example, the ^1^H NMR spectrum of the extract obtained using supercritical CO_2_ extraction of the spring harvest (scCO_2_Sp). The spectrum is dominated by the strong signal of the CH_2_ of alkyl chains. On the basis of the literature data, the following signals can be recognized: -CH_3_ of sterols (δ: 0.54 ppm), –CH_2_ of triterpenes (δ: 0.69 ppm). The signals of the fatty acids are visible in the range δ: 0.85–0.93 ppm (–CH_3_) and at δ: 1.6 ppm –CH_2_CH_2_COOH, at δ: 2.06, m –CH_2_-CH=CH-, and at δ: 2.23 m –CH_2_COOH, besides weaker signals of sesquiterpene lactones and triterpenes can be observed [5].

The ^1^H NMR spectrum of extracts obtained using supercritical CO_2_ from leaves collected in autumn (scCO_2_Au) is available in SI (Figure S1 in SI).

**Figure 4:**
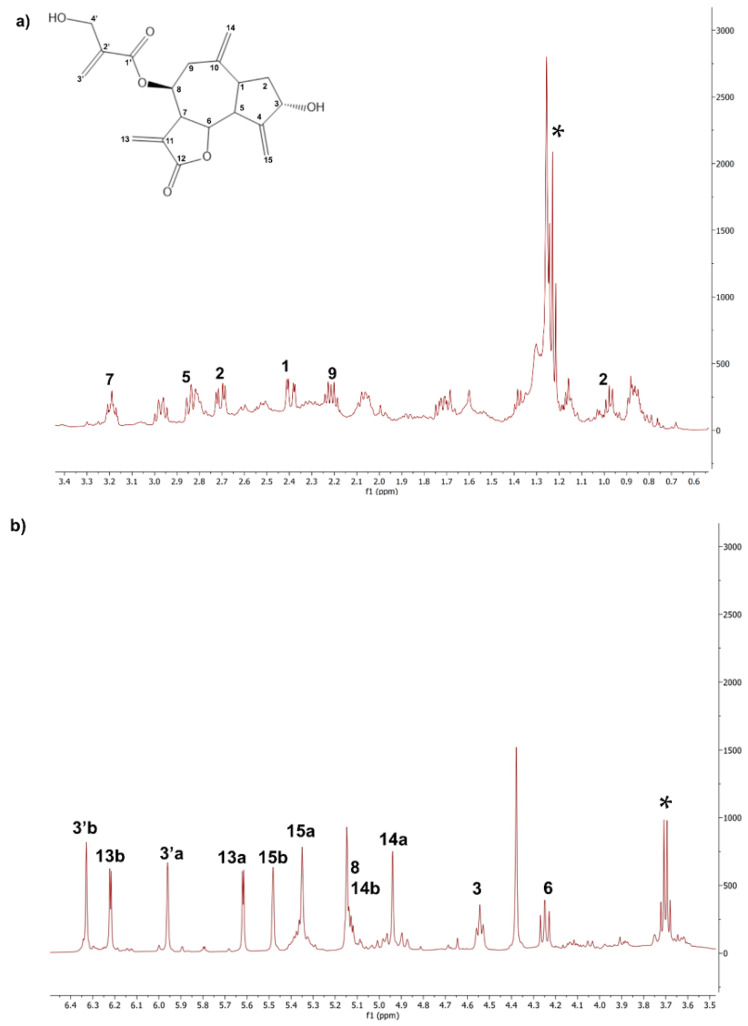
Details of the ^1^H-NMR spectrum of the CLE NaviglioSp. (**a**) Signals of cynaropicrin in the range of 0–3.4 ppm; (**b**) Signals of cynaropicrin in the range of 3.5–6.5 ppm. ^1^H-NMR (500 MHz, CDCl_3_): **1** δ: 2.43, dt; **2a** δ: 1.09, ddd; **2b** δ: 2.07, dt; **3** δ: 4.62, tt; **5** δ: 2.84, dd; **6**, δ: 4.27, dd; **7** 3.27, tt; **8** and **14b** δ: 5.17, ttt; **9a**–**b** δ: 2.25–2.45, dd; **13a** δ: 5.62, d; **13b**, δ: 6.25, d; **14a** δ: 4.96, d; **15a** δ: 5.43, t; **15b** δ: 5.52, t; **3′a** δ: 5.9, m; **3′b** δ: 6.35, m. * marks residual ethanol signals.

The ^1^H NMR spectrum of the CLEs obtained using the Naviglio^®^ technology (NaviglioSp) shows a significant presence of linolenic acid (δ: 0.9 ppm) and cynaropicrin. In addition, there are weak signals assignable to pheophytins, which give the characteristic green color to these extracts (δ: 4.49, –CH of pheophytins) [35]. Many signals of cynaropicrin, the most abundant sesquiterpene lactone, appear in the 6.5–3.5 ppm spectral region shown in Figure 4. For sake of completeness, both the ^1^H and ^13^C NMR whole scale spectra of all the extracts are reported in SI (Figures S1 and S2 in SI, respectively).

### 2.3. Evaluation of Cardoon Leaves Extracts on Rett Syndrome In Vitro Model

We previously established an in vitro model of Rett syndrome (RTT) [27] based on primary neuronal hippocampal cultures prepared using brains extracted from mice in which the MeCP2 gene was deleted (MeCP2^-/y^ mice) [26]. Starting from days-in-vitro 6 (DIV 6), cultured MeCP2^-/y^ neurons show atrophic morphology due to an arrest in neuronal growth [27]. Here, we tested the efficacy of CLEs in rescuing RTT neuronal development arrest using a miniaturized version of this assay optimized for reproducible and robust drug screening in 96-well plate format [40].

**Figure 5:**
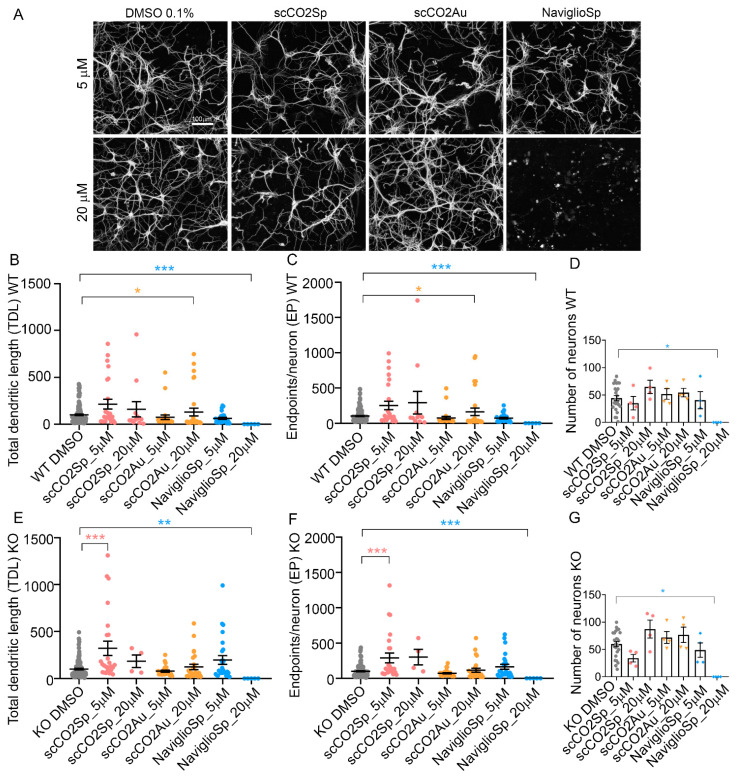
Treatments of neuronal cultures with cardoon extracts. (**A**) NeuriteQuant morphological analysis of TDL and Endpoints of DIV 6 hippocampal WT neurons, plated at the density of 160 cells mm^−2^ (*n* = 2). From left, WT neurons treated with DMSO 0.1% (control condition), scCO_2_Sp extract, scCO_2_Au extract, NaviglioSp extract. First line represents neurons treated at the concentration of 5 µM (in DMSO 0.1%), second line at 20 µM (in DMSO 0.1%). Scale bar: 100 µm. (**B**,**C**) Quantitative data of WT neurons, reporting the average TDL per neuron (µm) and the average number of endpoints per neuron. n = 22 images for a total of 2 independent biological replicates (cell cultures). (**D**) Number of WT neurons per each condition. (**E**,**F**) Quantitative data of MeCP2 KO neurons, reporting the average TDL per neuron (µm) and the average number of endpoints per neuron. n = 22 images for a total of 2 independent biological replicates (cell cultures). (**G**) Number of MeCP2 KO neurons per each condition. Kruskal–Wallis with Dunnett’s multiple comparisons test vs. DMSO conditions. *** *p* < 0.001, ** *p* < 0.01, * *p* < 0.05.

In a first set of experiments, Wild-Type (WT) and MeCP2^-/y^ (KO) neurons were incubated for 3 days, from DIV 3 to DIV 6, with the three different cardoon extracts (scCO_2_Sp, scCO_2_Au and NaviglioSp) at two concentrations (5 µM and 20 µM) expressed as a total mole concentration of the four considered bioactive molecules by considering a weight-averaged molecular weight. High content imaging microscopy analysis based on the NeuriteQuant software [31,40] (Figure 5A) was used to measure two morphological parameters, i.e., the total dendritic length (TDL; sum of the extension of all dendrites of a neuron in µm) and the number of dendritic endpoints (EP, a measure of the number of terminal branches of the dendrites of a neuron).

In WT neurons, the scCO_2_Au extract at 20 µM induced a significant increase of both TDL (133% ± 42.4 S.E.M; *p* = 0.0339) and EP (162% ± 53 S.E.M.; *p* = 0.0137) with respect to WT DMSO control (TDL = 100% ± 10.3 S.E.M.; EP = 100% ± 9.4 S.E.M) (Figure 5B,D). No significant effect was seen with the other extracts either at 5 or 20 µM concentrations (Figure 5B–D) while the NaviglioSp extract at 20 µM induced a significant neuronal toxicity, as indicated by the dramatic reduction in the morphological parameters and the number of neurons remaining in culture after the treatment (Figure 5B–D; *p* = 0.0005 for TDL, *p* = 0.0004 for EP, and *p* = 0.0154 for the number of neurons with respect to DMSO). In KO neurons, only the scCO_2_Sp extract at 5 µM showed a significant increase in TDL (321% ± 76 S.E.M; *p* = 0.0003) with respect to KO DMSO (TDL = 100% ± 9.8 S.E.M) (Figure 5E,F), while the other extracts were ineffective (Figure 5E,F) and the NaviglioSp extract at 20 µM induced a significant neurotoxicity (Figure 5E–G; *p* = 0.0028 for TDL, *p* = 0.0021 for EP, and *p* = 0.0094 for the number of neurons with respect to DMSO). In conclusion, only the cardoon scCO_2_Sp extract was able to induce a significant rescue of neuronal atrophy in RTT neurons, while the scCO_2_Au extract resulted to be active on WT neurons and the NaviglioSp was toxic on both WT and KO neurons.

**Figure 6:**
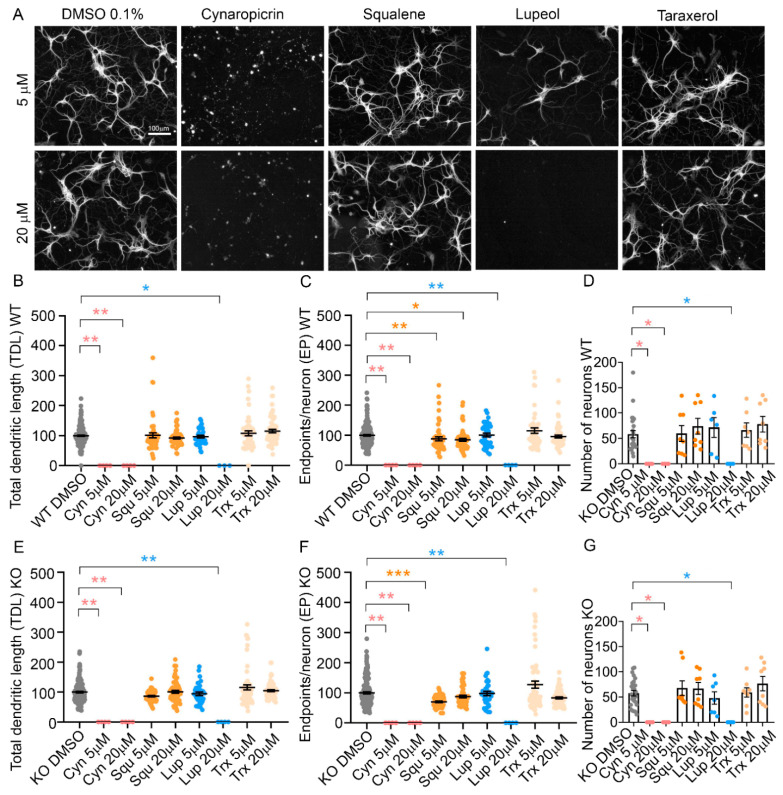
Treatments of neuronal cultures with bioactive molecules. (**A**) NeuriteQuant morphological analysis of TDL and Endpoints of WT hippocampal neurons at DIV 6 (n = 4). From left, WT neurons treated with DMSO 0.1% (control condition), cynaropicrin, squalene, lupeol, taraxerol. First line represents neurons treated at the concentration of 5 µM (in DMSO 0.1%), second line at 20 µM (in DMSO 0.1%). Scale bar: 100 µm. (**B**,**C**) Quantitative data of WT neurons, reporting the average TDL per neuron (µm) and the average number of endpoints per WT neuron n = 44 images for a total of 4 independent biological replicates (cell cultures). (**D**) Number of WT neurons per each condition. (**E**,**F**) Quantitative data of MeCP2 KO neurons, reporting the average TDL per neuron (µm) and the average number of endpoints per neuron. n = 44 images for a total of 4 independent biological replicates (cell cultures). (**G**) Number of MeCP2 KO neurons per each condition. Kruskal–Wallis with Dunnett’s multiple comparisons test vs. DMSO conditions. *** *p* < 0.001, ** *p* < 0.01, * *p* < 0.05.

In a second set of experiments, we tested the efficacy of individual pharmacologically active ingredients present in the different cardoon extracts, namely cynaropicrin, squalene, lupeol and taraxerol (Table 1). Each bioactive ingredient was incubated on WT and KO hippocampal neurons for 3 days from DIV 6, at the concentration of 5 or 20 µM, and TDL and EP were measured as experimental read-out (Figure 6A). No individual bioactive compound was able to replicate the positive effect on TDL and EP observed with the whole extracts of scCO_2_Sp and scCO_2_Au in KO or WT neurons (Figure 5B,C,E,F). However, we observed a significant neurotoxic effect of cynaropicrin at both 5 and 20 µM, and of lupeol at the higher concentration of 20 µM (Figure 6B–G). In particular, cynaropicrin 5 µM (*p* = 0.0039), and 20 µM (*p* = 0.0039) for WT TDL; cynaropicrin 5 µM (*p* = 0.0019), 20 µM (*p* = 0.0019) for WT EP; cynaropicrin 5 µM (*p* = 0.0263) for the number of WT neurons; cynaropicrin 20 µM (*p* = 0.0263) for the number of WT neurons; Squalene 5 µM (*p* = 0.0081), 20 µM (*p* = 0.0107) for WT EP; lupeol 20 µM (*p* = 0.0197) for WT TDL, (*p* = 0.0019) for WT EP, (*p* = 0.0097) for the number of WT neurons; cynaropicrin 5 µM (*p* = 0.0044), 20 µM (*p* = 0.0044) for KO TDL; cynaropicrin 5 µM (*p* = 0.0019), and 20 µM (*p* = 0.0019) for KO EP; cynaropicrin 5 µM (*p* = 0.0148) for the number of KO neurons; cynaropicrin 20 µM (*p* = 0.0148) for the number of KO neurons. Squalene 5 µM (*p* = 0.0001) for KO EP; lupeol 20 µM (*p* = 0.0044) for KO TDL, and *p*= 0.0019 for KO EP, *p* = 0.0148 for the number of KO neurons. These results support the conclusion that single bioactive ingredients are not sufficient to induce the positive effects of the whole extracts but are able to fully exert the toxic effects.

The use of primary neuronal cultures for screening large libraries of small molecules is a standard in the pharmaceutical industry and has been widely described in the literature for in vitro models of neurodegenerative disorders such as Alzheimer’s, Parkinson’s, Huntington’s diseases, and Amiotrophic Lateral Sclerosis [41,42,43]. More recently, similar approaches have also been undertaken for neurodevelopmental diseases such as Rett and Fragile-X syndromes [44]. Major limitations with these in vitro models concern, first of all, the mutation present in the mouse model used for neuronal cultures, which not necessarily corresponds to the actual mutation present in the majority of patients. This is the case for instance of Rett syndrome in which multiple mutations exist (more than 100, with 8 being the most frequent ones) leading to a variety of symptoms and disease severity [45]. In addition, there is a wide debate concerning the developmental stage at which the drugs screening should be carried out to obtain valuable results. Reasons of convenience have suggested that we should carry out the majority of drug screenings at an early stage of development, in order to obtain highly reproducible cultures within the shortest possible time, leading to obvious economies in terms of time and people employed. However, the necessity of an appropriate matching between drug screening and the onset of the most important cellular defects and aberrant processes has to be taken into consideration. In our case, we previously described in detail the various stages of development in vitro of a neuron from mice deleted of the MecP2, mimicking Rett syndrome [27]. More specifically, we showed that significant growth arrest in terms of reduced total dendritic length and number of secondary dendrites can already be quantified at DIV 6, while synaptogenesis is apparently normal at this stage, becoming clearly reduced at later stages, in particular from DIV 9 onward [27]. Collectively, these previous studies indicate the suitability of the use of primary in vitro cultures for drug screening. As regard to the effective bioavailability of plant extracts in the brain, some information is available but some in vivo studies have found rapid targeting into the brain upon oral or systemic delivery for flavonoids [46] and other polyphenol metabolites [47]. 

Taraxerol, squalene and lupeol found in cardoon leaves extracts are synthesized in plants through a common biosynthetic pathway called the mevalonate pathway, starting from Acetyl-CoA as a primary source. Specifically, squalene is the primary precursor for the synthesis of triterpenoids including taraxerol, and lupeol by taraxerol synthase [48]. Most interestingly, in mammalian cells squalene is also the precursor of cholesterol through the squalene epoxidase pathway, which is defective in Rett syndrome, leading to a significant reduction in cholesterol availability in the brain [49]. Lupeol has shown neuroprotective properties in animal models of neurodegenerative disorders [50], traumatic brain injury or ischemia [51,52] and, similar to taraxerol, also to have anti-inflammatory and pro-neurotrophic actions in vitro [53,54]. Comparatively less information is available for squalene, which was shown to counteract neuronal cell death in an in vitro model of Alzheimer’s-like injury [55]. Although in our experiments we used concentrations of taraxerol, squalene and lupeol within a range comparable to the one used in the in vitro studies cited above [53,54,55], we did not detect significant neuroprotective activity against neurodevelopmental deficits in our in vitro Rett syndrome model. Differences in cellular models adopted, i.e., mouse hippocampal neurons in our study versus rat hypothalamic or cortical neurons, or an immortalized cell line in the other studies, may provide a first explanation for the different results obtained. On the other hand, our results suggest that the coexistence of deficits in multiple metabolic pathways, which is typical of syndromic disorders such as Rett syndrome, may require a coordinated polypharmacological approach against multiple targets, which may be achieved only with a complex mixture of bioactive components.

## 3. Materials and Methods

Solvents, reagents, and standard solutions of bioactive molecules were purchased from Merck KGaA, Darmstadt (Germany) and used as received if not otherwise specified. 

Cardoon leaves were kindly provided by Novamont (Novara, Italy) and were taken from the cultivation of *Cynara cardunculus* var. *altilis* in Terni in spring and autumn 2020. 

### 3.1. Preparation of Leaves Samples

The cardoons were pre-treated by separating leaves from the stalks. Cardoon samples were obtained from a biorefinery crop, which was harvested in large quantities in a precise period of the year. Therefore, in this study we did not consider individual plants. It must be underlined that in our previous study we demonstrated that the yield of extractions conducted on different samples and aliquots of the same batch of plants has an error of less than 5% [16].

The material was cut by means of garden scissors in square pieces of about 1 cm, which were temporarily stored under vacuum in plastic bags (about 200 g each) at −20 °C. The material used for scCO_2_ extraction was dried by means of a first step of lyophilization for 48 h. The treatment allowed us to remove about 82–85% of water, calculated by weight difference. A second treatment in an oven at 40 °C for 48 h led to the removal of a further 0.5% of water, with respect to the lyophilized samples. The dried samples were temporarily stored under vacuum in plastic bags at +3 °C before analysis. 

Cardoon leaves addressed to the Naviglio^®^ extractor were separated from the fresh plants and stored in vacuum-sealed plastic bags (about 200 g each) at −20 °C. For extract preparation, cardoon leaves were thawed at room temperature and cut into 1 cm pieces.

### 3.2. Extraction Methods

1. Supercritical CO_2_ extraction: 6–8 g of dried leaves was loaded in a 100 mL extractor. The scCO_2_ extraction system [56] was composed by a Separex SFE 20 unit (heated stainless-steel extractor 100–200 mL, laminating valve Tescom 26-1000, heated collecting chamber) connected to a liquid CO_2_ cylinder, a high-pressure pump Lewa EKM210V1 and an EL-FLOW Bronkhorst flowmeter. Conditioning was performed for 30 min and then the extraction was started by turning on the pump with a carbon dioxide flowrate of 120 L h^−1^ for 2 h at 45 °C and 225 bar. The extracts were collected by dissolving the oily mixture in diethyl ether (<1 mL). The extraction yields were 3.7 and 2.0 % (*w w*^−1^) in the case of autumn- and spring-harvested plants, respectively.
2. Naviglio^®^ method: Filter bags (porosity of 100 μm) were filled with 40 g of cut cardoon leaves and then inserted into the extraction chamber of the Naviglio^®^ extractor (500 cm^3^ capacity). Extractions were conducted using 625 mL of anhydrous ethanol at 25 °C (9 bar, static phase 2 min; dynamic phase 2 min, with 12 s stop piston). Liquid samples were collected at 24 h. The extraction yield was 4.8 % (*w w*^−1^). The cardoon leaf extracts (CLE) were stored at 4 °C until analysis. Ethanol was chosen as a solvent for phenols extraction, as described in the literature [16].

### 3.3. Characterization of Extracts by Means of NMR

The ^1^H and ^13^C NMR analysis was performed by dissolving 10 mg of CLE in 0.7 mL of deuterated chloroform. The NMR spectra were acquired at 25 °C by a Varian VNMRS 500 NMR spectrometer (11.74 T) operating at 500 MHz for proton and 125 MHz for carbon, using 256 scans for proton and 16000 scans for carbon, interleaved by 7.7 s for proton and 2.05 s for carbon, with 45° pulses, employing a spectral width of 8012.8 Hz for proton and 31250 Hz for carbon over 32 K complex points. The signals were assigned according to the literature [5,57]. 

### 3.4. Quantification of Cynaropicrin, Squalene, Taraxerol and Lupeol

The bioactive molecules were quantified by GC-MS (Shimadzu GC-MS-QP2020). Calibration curves were constructed by using commercial standards of cynaropicrin (SI, Figure S4), squalene (SI, Figure S5), taraxerol (SI, Figure S6) and lupeol (SI, Figure S7) and dodecane as internal standard. The analysis was performed on samples prepared by dissolving 0.7 mg of each CLE in 1 mL of diethyl ether. The separation was obtained on a 30 m × 0.25 mm fused-silica capillary column (SLB5ms) coated with a 0.25 μm film of poly(5% phenyl, 95% dimethyl siloxane). The mass spectrometer was set to scan the *m*/*z* range 33–700. Samples were injected (1 μL) with a splitting ratio 1:20 and the injector temperature was set to 280 °C. The column oven was initially set at 50 °C and maintained for 2 min after the injection, followed by a temperature ramp (8 °C min^−1^) up to 250 °C followed by a second ramp (3 °C min^−1^) up to 280 °C. The total analysis time was 63.33 min [29]. 

### 3.5. Mice Strain and Genotyping

The animal use was approved by the Italian Ministry of Health (authorization n. 693/2021-PR issued on Sept. 6th, 2019), in conformity with the Italian legislation D.Lgs 116/92. Animals were housed under standard conditions, in ventilated cages, in a 12/12 h light/dark cycle with food and water ad libitum. Wild-Type (WT) C57BL/6 male mice (Charles River Laboratories, Calco, LC, Italy) were crossed with C57BL/6 female heterozygous for the deletion of exon 3 and 4 in MeCP2 gene1 [26]; MeCP2^−/+^, B6.129P2(C)-Mecp2tm1.1Bird/J, stock: 003890, Jackson Laboratories, Bar Harbor, Maine) to obtain Wild-Type (MeCP2^+/y^, WT) and Knock-Out (MeCP2^−/y^, KO) male mice.

The mice genotype was determined using DNA extracted from tails using KAPA Express kit (EXPEXTKB; Roche). Polymerase Chain Reaction (PCR) was performed using KAPA2G Fast DNA polymerase with the following primers: 5′- AAATTGGGTTACACCGCTGA-3′ (Common Forward 9875, Jackson Laboratory), 5′-CTGTATCCTTGGGTCAAGCTG-3′ (Wild-Type Reverse oIMR7172, Jackson Laboratory), 5′- CCACCTAGCCTGCCTGTACT-3′ (Mutant Reverse 9877, Jackson Laboratory). PCR reaction was performed in a final volume of 25 µL set as follows: initial denaturation at 95 °C for 3 min then, 95 °C for 20 s, 58 °C for 20 s, 72 °C for 20 s (35 cycles) and final elongation at 72 °C for 2 min.

### 3.6. Culture of Hippocampal Primary Neurons (HPN)

**Figure 7:**
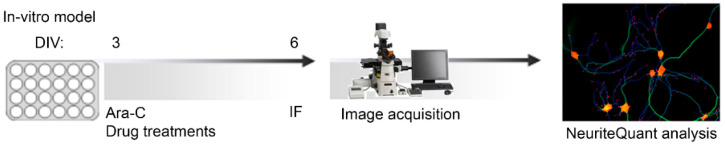
Workflow for phenotypic screening. Primary hippocampal neurons were plated at DIV 0 in 96 multi-well, and at DIV 3 were added D-Arabinofuranoside (ARA-C, Sigma) and treatments with extracts or bioactive molecules. Neurons were fixed in PFA 4% at DIV 6 and immunofluorescence (IF) was performed with anti-MAP2 (red), and anti-NeuN (green). Images were acquired using Nikon Eclipse Ti-E epifluorescence live imaging microscope equipped with Nikon DS-Qi2 camera, using 10× objective. Eleven random fields (3.0 × 3.0) per well were acquired and analyzed individually. Total Dendritic Length (TDL) and the number of Endpoints per neuron (EP) were measured with NeuriteQuant software, implemented as a plugin of ImageJ [58].

Primary hippocampal neuronal cultures were prepared from P0 and P1 male mice, both Wild-Type (MeCP2^+/y^, WT) and Knock-Out (MeCP2^-/y^, KO), according to Baj et al., 2014 [27]. In brief, mice were sacrificed by decapitation, hippocampi were extracted (under bright field microscope) and collected in cold Hank’s balanced salt solution HBSS (sodium bicarbonate (NaHCO_3_) 4.2 mM, Hank’s salt powder 0.952%, HEPES 12 mM, D-Glucose, 200 μM kynurenic acid, BSA, magnesium sulphate (MgSO_4_), Sigma, St. Louis, MO, USA). The tissue digestion was performed by adding 0.25% Trypsin (Euroclone, Milan, Italy) for 8 min at 37 °C. The enzymatic digestion was blocked with 1.5 mL of Dulbecco’s Modified Eagle Medium high glucose (DMEM, Euroclone, Milan, Italy), supplemented with 10% Fetal Bovine Serum (FBS, Euroclone, Milan, Italy) and penicillin-streptomycin (P/S, Euroclone, Milan, Italy). The tissue was centrifugated at 800 rpm for 5 min at room temperature (25 °C) and then resuspended with 1 mL of DMEM  +  10% FBS and mechanically triturated. Cells were counted with the dye exclusion method using Trypan Blue (Sigma) in the Burker chamber (Eppendorf), obtaining 800,000–900,000 cells from each mouse. Cells were plated in 96 MW plates (Sarstedt, Nümbrecht, Germany), previously treated with 0.2% Poly-L-Ornithine (PORN, Sigma) to allow the attachment of cells. Cells were seeded at the density of 160 cell mm^2^ and were grown at 37  °C and 5% CO_2_ in Neurobasal (Invitrogen, Waltham, MA, USA) supplemented with 2% B-27 (Invitrogen), 1 mM L-glutamine and 1% penicillin-streptomycin. Cell medium was changed at DIV 3 including Cytosine β-D-arabinofuranoside (Ara-C, Sigma) at the final concentration of 2.5 µM to inhibit proliferation of non-neuronal cells. Cells were maintained in culture until DIV 6 (Figure 7). 

### 3.7. Treatments

Treatments with extracts or bioactive molecules were performed for 3 days, from DIV 3 to DIV 6 (Figure 7). The extracts scCO_2_Au, scCO_2_Sp and NaviglioSp were tested at concentrations 5 µM and 20 µM obtained as follows: a 5 mM solution in 100% DMSO was firstly diluted 1:100 and then 1:10 to reach a 5 μM solution of extract in culture medium containing ultimately, upon dilution, 0.1% of DMSO. The same dilution steps were applied to the 20 mM solution to reach a final 20 μM concentration (n = 22 images for a total of 2 independent biological replicates). 

The same concentrations were used to test the single bioactive molecules: cynaropicrin (Cyn), squalene (Squ), lupeol (Lup) and taraxerol (Trx) (n = 22 images for a total of 4 independent biological replicates). In a previous study, we provided evidence that the number of neurons per mm^2^ and the average TDL in cultures treated with DMSO 0.1% are not significantly different with respect to the untreated condition [40]. Thus, DMSO 0.1% represents the control condition of each treatment. 

### 3.8. Immunofluorescence

Hippocampal primary cultures were fixed at DIV 6 using 4% Paraformaldehyde (PFA, Sigma) in PBS 1x for 15 minutes at room temperature, then washed with PBS 1x and permeabilized using PBS-Triton 0.1% for 15 minutes. In order to avoid unspecific bindings, the blocking solution was added with PBS-Triton 0,1% and 2% Bovine Serum Albumin (BSA, Sigma) for 15 min. Primary antibodies (Table 2) were specific to detect the Microtubule-associated protein 2 (MAP2), an abundant microtubule-associated protein implicated in the formation and outgrowth of neuronal processes (dendrites and axons), and to detect Neuronal nuclei (NeuN), present specifically in the nuclei of mature neurons [50,59]. Primary antibodies were diluted in blocking solution and cells were incubated for 1:30 h at room temperature in a dark humified chamber and oscillated in a rocker. Cells were washed with PBS1x (5 min/wash) and then incubated with the secondary antibodies anti-rabbit IgG Alexa Fluor568 (Invitrogen, A10042) and anti-mouse IgG Alexa Fluor488 (Invitrogen, A11001) for 1:30 h at room temperature, in a dark humified chamber and on a rocker. Both secondary antibodies were diluted 1:1000. Cells were washed with PBS-Triton 0.1% and then with PBS 1x (5 min/wash) and incubated with Hoechst 33342 (10 mg ml^−1^, Sigma) at the dilution 1:1000 (final concentration 10 μg ml^−1^) in PBS1x for 7 min. Then, Hoechst was washed with PBS1x.

### 3.9. Image Acquisition

Images were acquired at the Nikon Eclipse Ti-E epifluorescence live imaging microscope equipped with a motorized stage and a Nikon DS-Qi2 camera (CMOS sensor, 16.25-megapixel, 14 bit gray levels). Acquisitions were performed using the software Nis-Elements 4.60 with the module “JOBS” for automated imaging. Per single well, 11 random images (3.0 × 3.0 fields) were acquired using the 10x objective. Image size was 14 bit-1636 × 1088 pixels, which corresponds to 1.440 mm × 0.957 mm. The number of seeded neuronal and non-neuronal cells were respectively obtained by automatic counting of NeuN-positive neurons and Hoechst-positive cells with the “Objective-analyser” plugin for Nis-Element 4.60. Image acquisition parameters: 900 ms of exposure time for MAP2, 2 s of exposure time for NeuN, 20 ms of exposure time for Hoechst, filters at 1.

### 3.10. NeuriteQuant Morphological Analysis

Each image was analyzed individually with the NeuriteQuant open-source software, which is able to perform the fast and accurate analysis of a large set of images [44]. For each image, the following parameters were measured:

1. Total Dendritic Length: sum of the length of all the dendrites present in one image;
2. Number of Endpoints per neuron: number of terminal points counted at the end of visible dendritic staining (MAP2).

The analysis is highly sensitive and independent from signal intensity, allowing the detection of both neurites characterized by a strong signal or by a weaker signal intensity. In order to perform the analysis, four parameters have to be set:

1. Neurite detection width: 12;
2. Neurite detection threshold: 8;
3. Neurite clean-up threshold: 170;
4. Neuronal cell body detection: 300.

Before starting the morphological analysis with NeuriteQuant, we used the Enhance Local Contrast (CLAHE) plugin of ImageJ to enhance the immunofluorescence signal to obtain a better contrast of the image.

### 3.11. Statistical Analysis

All the statistical data analysis and data representation were performed on Prism 8.0 software (Graphpad), while data organization was performed using Microsoft Excel 2018 (build 14326.20508) (Office). All data were checked using the Shapiro–Wilk normality test. One-way ANOVA for multiple comparisons to compare more than two groups was performed when data were normally distributed. When data were not normally distributed, the statistical difference was calculated using the Kruskal–Wallis test comparing more than two groups. Outlier detection was performed using Grubb’s test with GraphPad software.

## 4. Conclusions

The demonstration that the reactivation of the MECP2 gene in a mouse model can rescue large part of the Rett syndrome-like phenotypes has revealed that the disease is reversible, paving the way towards the search for new treatments [58,60]. Recent studies have discovered that lipid metabolism is perturbed in the brain and in the liver of mouse models of Rett syndrome, and this deficit, and other general symptoms of the disease, were rescued by regulating the cholesterol synthesis pathway either genetically or pharmacologically [45,49]. Hence, it is very intriguing that the scCO_2_ hydrophobic extracts are rich in squalene, one of the intermediates of cholesterol synthesis, even though in our experiments pure squalene was not sufficient to revert dendritic atrophy in vitro, suggesting that the whole extract provides a better neuroprotective effect than single molecules. In conclusion, the significant rescue of the aberrant phenotype of RTT neurons obtained with the hydrophobic scCO_2_ extract of leaves from *Cynara cardunculus* var *altilis* harvested in spring warrants further studies to characterize in detail the composition of the extracts and further investigate the mechanism of action in Rett syndrome. Overall, this study indicates that it is crucial to design optimal extraction procedures, both in terms of selection of harvesting period and extractive technologies, to maximize the pharmacological potential of bioactive extracts. 

## Figures and Tables

**Table 1:** Composition of the principal bioactive molecules in the three solutions of cardoon leaves extracts.

	Composition * in scCO_2_Au (*w w*^−1^)	Composition * in scCO_2_Sp (*w w*^−1^)	Composition * in NaviglioSp (*w w*^−1^)
Cynaropicrin	0.040	0.020	0.230
Squalene	0.480	0.390	0.110
3ß-Taraxerol	0.070	0.030	<0.001
Lupeol	0.090	0.050	0.010

* Composition expressed as *w w*^−1^ of each bioactive molecule/1 mg of extract.

**Table 2:** Primary antibodies used.

Primary Antibody	Species	Dilution	Company	Code
Anti-MAP2	Rabbit	1:500	Genetex	GTX50810
Anti-NeuN	Mouse	1:500	LS-Bio	LS-C312122-100

## Data Availability

The data presented in this study are available on request from the corresponding author.

## Supplementary Materials

The following supporting information can be downloaded at: https://www.mdpi.com/article/10.3390/molecules27248772/s1, Figure S1: ^1^H-NMR (500 MHz, CDCl_3_) spectrum of the CLE (a) scCO_2_Au; (b) NaviglioSp 1; Figure S2: ^13^C NMR spectra from samples: (a) scCO_2_Au, (b) scCO_2_Sp, (c) NaviglioSp; Figure S3. GC-MS chromatogram and fragmentation of bioactive molecules. (a) cynaropicrin; (b) squalene, (c) 3ß-taraxerol and (d) lupeol; Figure S4. GC-MS calibration curve of cynaropicrin; Figure S5. GC-MS calibration curve of Squalene; Figure S6. GC-MS calibration curve of Taraxerol; Figure S7. GC-MS calibration curve of Lupeol. References [5,61] are cited in the supplementary materials.

## Sample Availability

Samples of the compounds are available from the authors.
