# Supplementary material for: Neuroprotective Properties of Cardoon Leaves Extracts against Neurodevelopmental Deficits in an In Vitro Model of Rett Syndrome Depend on the Extraction Method and Harvest Time
Source: Molecules. 2022 Dec 10;27(24):8772. doi: 10.3390/molecules27248772 (PMC9783035; doi:10.3390/molecules27248772)
Supplement: Supplementary file 1 [file molecules-27-08772-s001.zip › molecules-2021601-supplementary.pdf]

Neuroprotective properties of cardoon leaves extracts against neurodevelopmental deficits in an in vitro model of Rett syndrome depend on the extraction method and harvest time

Mariachiara Spennato [1], Ottavia Maria Roggero [2], Simona Varriale [3], Fioretta Asaro [1], Angelo Cortesi [4], Jan Kašpar [1], Enrico Tongiorgi [2], Cinzia Pezzella [3] and Lucia Gardossi [1,*]

[1] Department of Chemical and Pharmaceutical Sciences, University of Trieste, Via L. Giorgieri 1, 34127 Trieste, Italy
2 Department of Life Sciences, University of Trieste, Via L. Giorgieri 5, 34127 Trieste, Italy
3 Department of Chemical Sciences, University Federico II of Naples, Via Cinthia, 4, 80126 Napoli, Italy
4 Department of Engineering and Architecture, University of Trieste, Via Alfonso Valerio 6/A, 34127 Trieste, Italy
* Correspondence: gardossi@units.it

Supplementary Information (SI)

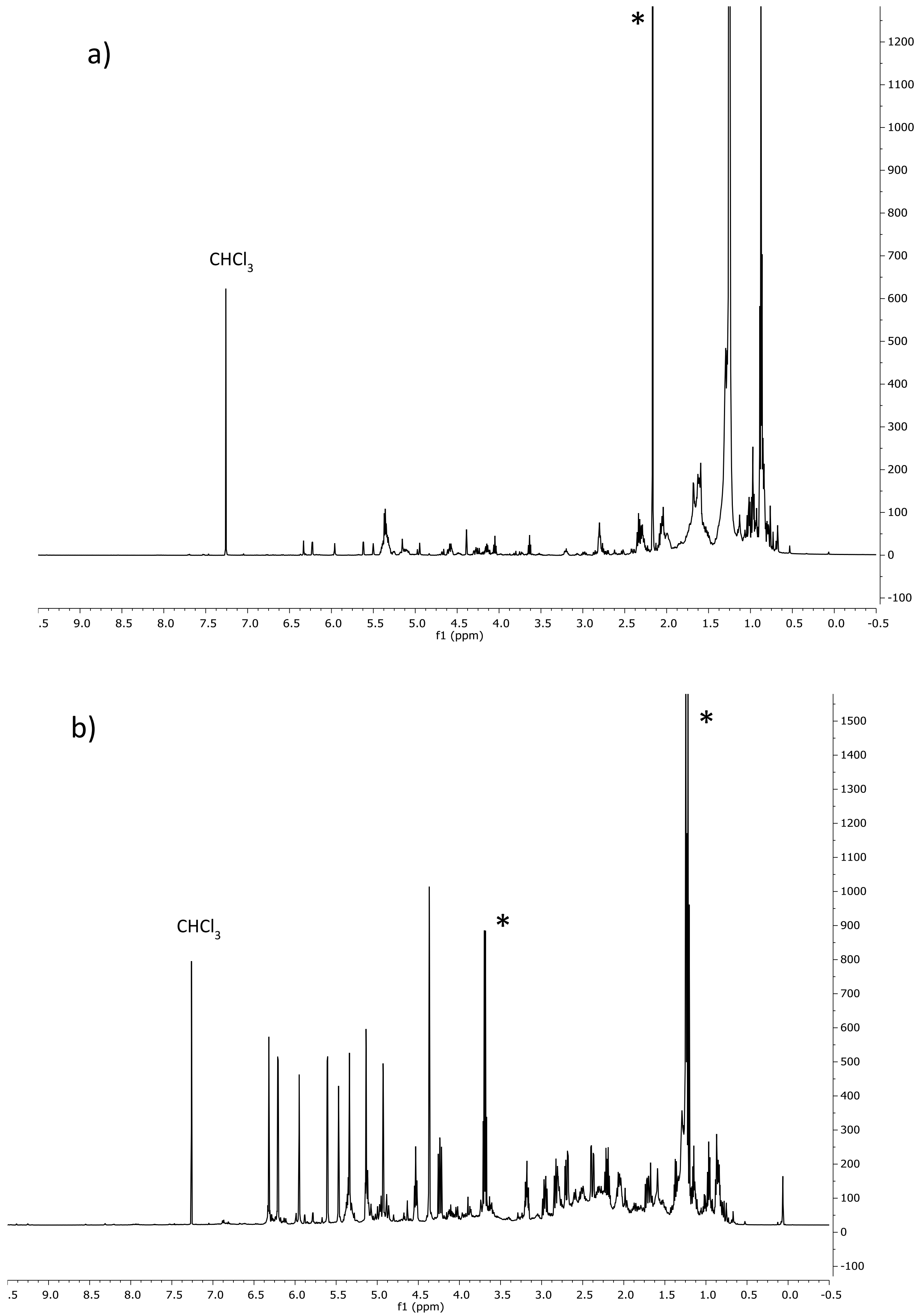

Figure S1. ^{1}H-NMR (500 MHz, $CDCl_3$) spectrum of the CLE a) scCO2Au; b) NaviglioSp. δ: 0.53 -CH_3 of sterols; δ: 0.71 -CH_2 of triterpenes; δ: 0.85-0.96 -CH_3 alkyl chains; δ:1.30-1.22 -CH_2 of alkyl chains; δ: 1.59 -$C\underline{H}_2CH_2COOH$ of fatty acids; δ: 2.16 -$C\underline{H}_2$-CH=CH- ; δ: 2.21 -$C\underline{H}_2COOH$ of fatty acids; δ: 4.61-4.54 signals of lupeol; δ: 4.65-4.67 signal of taraxerol, δ: 4.27, 4.48,4.94,5.14,6.33,6.22,5.95,5.61,5,49 typical signals of cynaropicrin as reported in the main manuscript; * denotes solvents (Acetone-Ethanol, respectively in a) and b)).

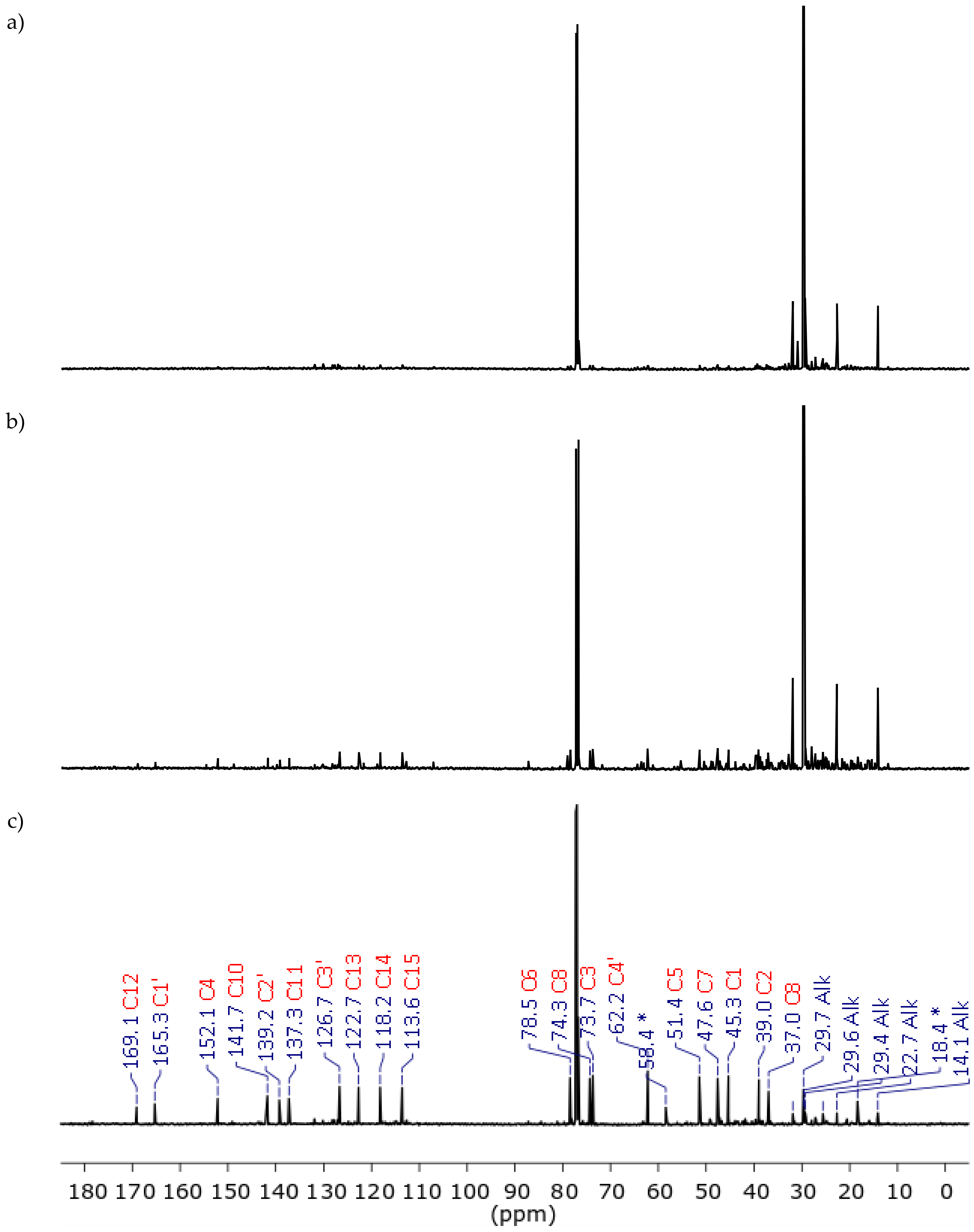

Figure S2. ^{13}C NMR spectra from samples: a) scCO2Au, b) scCO2Sp, c) NaviglioSp. On the last spectrum is reported the assignment of the signal of cynaropicrin and paraffins, carried out on the basis of literature data [1,2]. The asterisks mark the peaks of residual EtOH.

a) Cynaropicrin

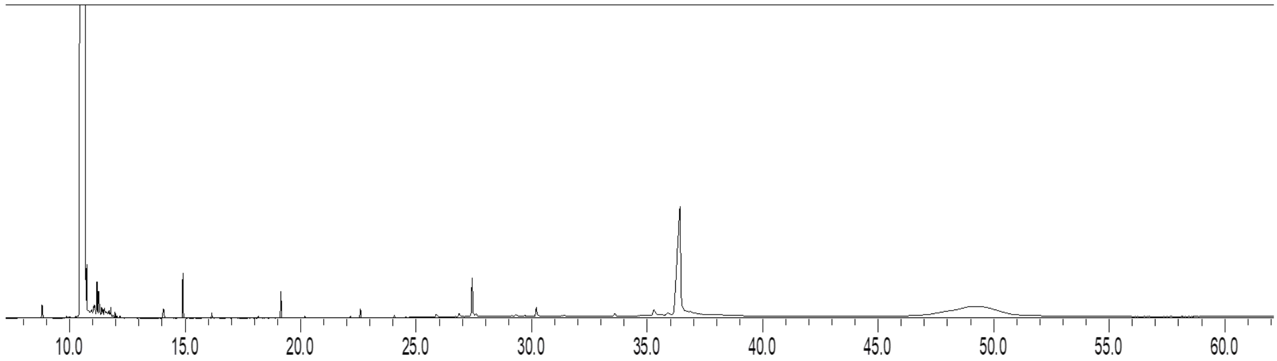

Peak Report TIC

Peak#	R.Time	I.Time	F.Time	Area	Area%	Height	Height%	A/H	Mark	Name
1	10.675	10.233	10.833	736963593	91.77	53775329	83.82	13.70	MI	Dodecane, 2,6,11-trimethyl-
2	14.058	13.850	14.267	1384516	0.17	406226	0.63	3.41	MI	Cycloheptasiloxane, tetradecamethyl-
3	14.899	14.700	15.100	3183564	0.40	1942007	3.03	1.64	MI	Butylated Hydroxytoluene
4	19.146	18.917	19.383	2431715	0.30	1145068	1.78	2.12	MI	Tributyrin
5	27.419	27.283	27.700	5524401	0.69	1636659	2.55	3.38	MI	Andrographolide
6	30.198	30.067	30.383	1360961	0.17	381953	0.60	3.56	MI	m-Camphorene
7	35.288	35.100	35.467	1456665	0.18	219306	0.34	6.64	MI	1H-Cyclopropa[3,4]benz[1,2-e]azulene-5,7b,9
8	36.415	36.017	37.050	50729625	6.32	4648014	7.25	10.91	MI	Cynaropicrin
				803035040	100.00	64154562	100.00			

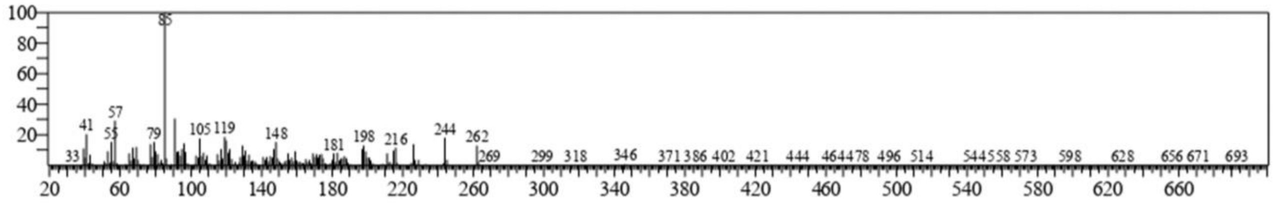

b) Squalene

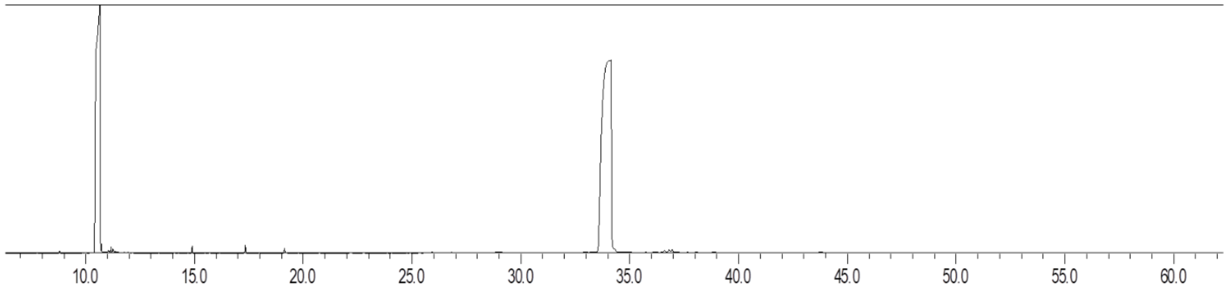

Peak Report TIC

Peak#	R.Time	I.Time	F.Time	Area	Area%	Height	Height%	A/H	Mark	Name
1	10.642	10.217	11.050	622502702	34.89	50778812	56.14	12.26	MI	Dodecane
2	28.950	28.567	29.583	3235480	0.18	178254	0.20	18.15	MI	D:A-Friedooleanan-3-ol, (3.alpha.)-
3	34.142	33.133	34.650	1158575312	64.93	39493858	43.66	29.34	MI	
				1784313494	100.00	90450924	100.00			

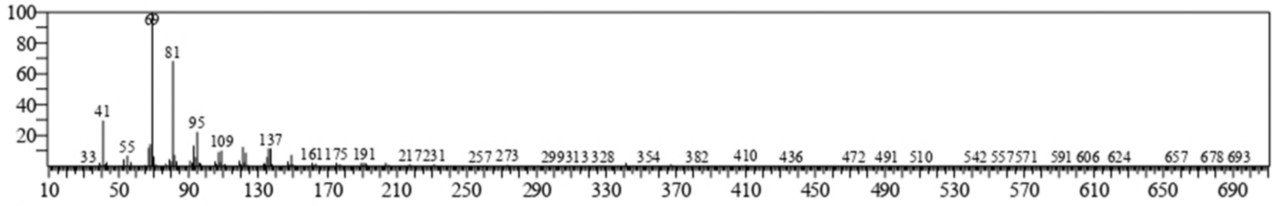

c) Taraxerol

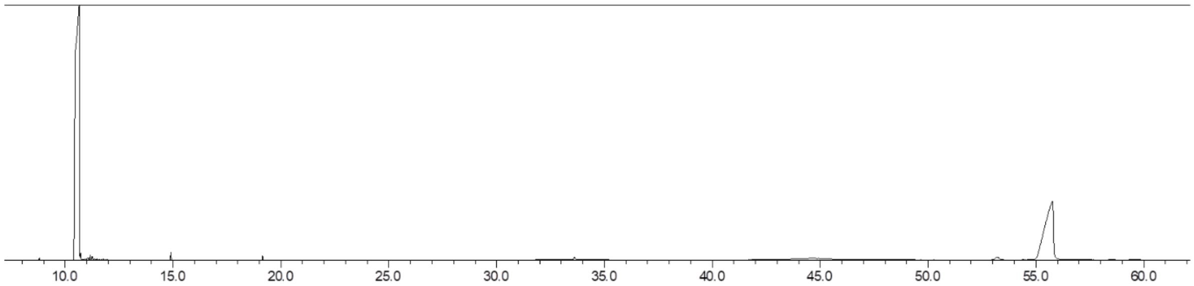

Peak Report TIC

Peak#	R.Time	I.Time	F.Time	Area	Area%	Height	Height%	A/H	Mark	Name
1	10.642	10.217	10.933	621463513	65.41	50729672	80.65	12.25	MI	Dodecane
2	53.197	52.767	53.600	7580568	0.80	536591	0.85	14.13	MI	D-Friedoolean-14-en-3-one
3	55.760	54.633	56.200	321051979	33.79	11633306	18.50	27.60	MI	Taraxerolo
				950096060	100.00	62899569	100.00			

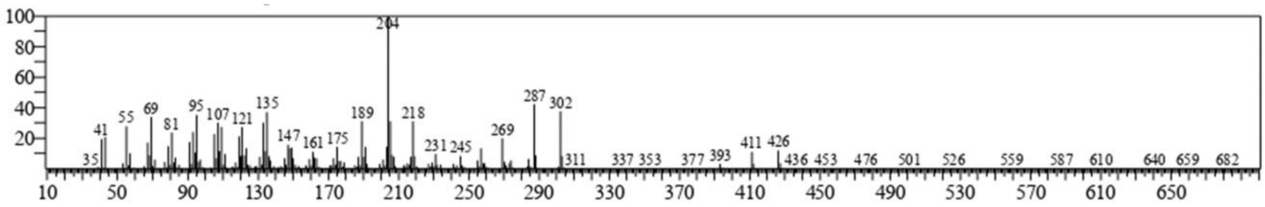

d) Lupeol

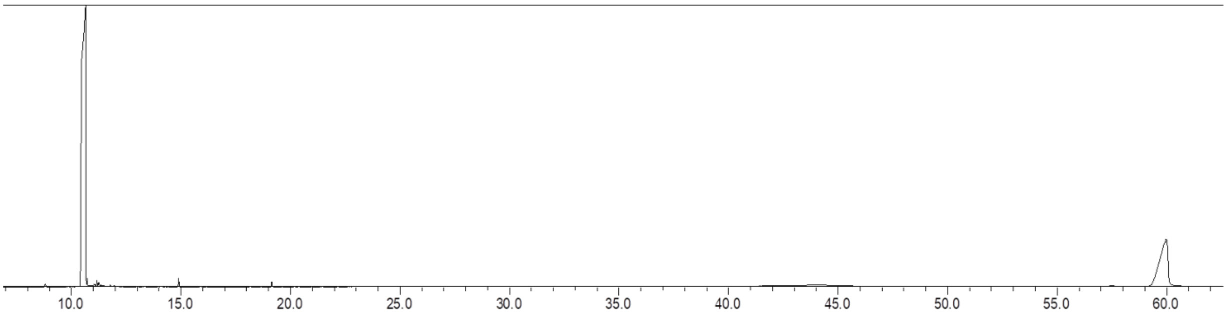

Peak Report TIC

Peak#	R.Time	I.Time	F.Time	Area	Area%	Height	Height%	A/H	Mark	Name
1	10.642	9.867	10.850	622601923	73.41	51251486	85.59	12.15	MI	Dodecane
2	59.970	58.750	60.717	225557845	26.59	8631350	14.41	26.13	MI	Lupeol
				848159768	100.00	59882836	100.00			

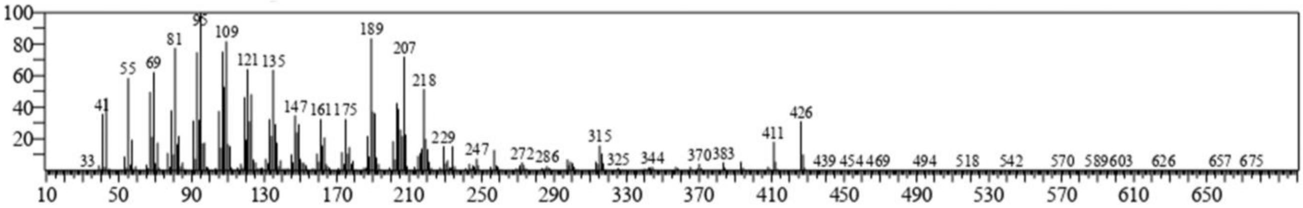

Figure S3. GC-MS chromatograms and fragmentations of bioactive molecules. a) cynaropicrin; b) squalene, c) 3ß-taraxerol and d) lupeol

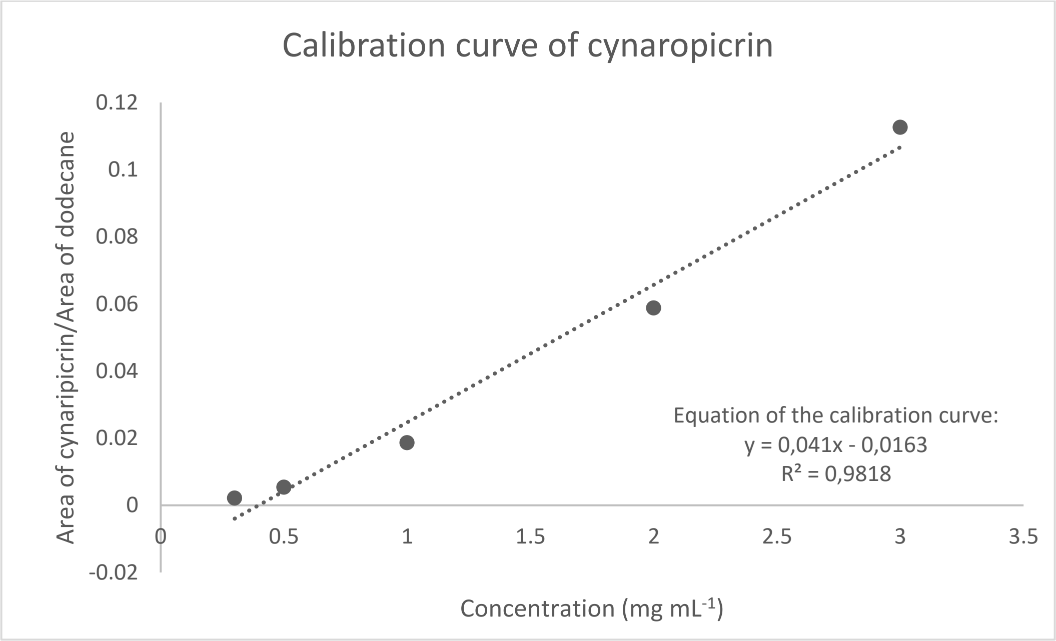

Figure S4. GC-MS calibration curve of Cynaropicrin

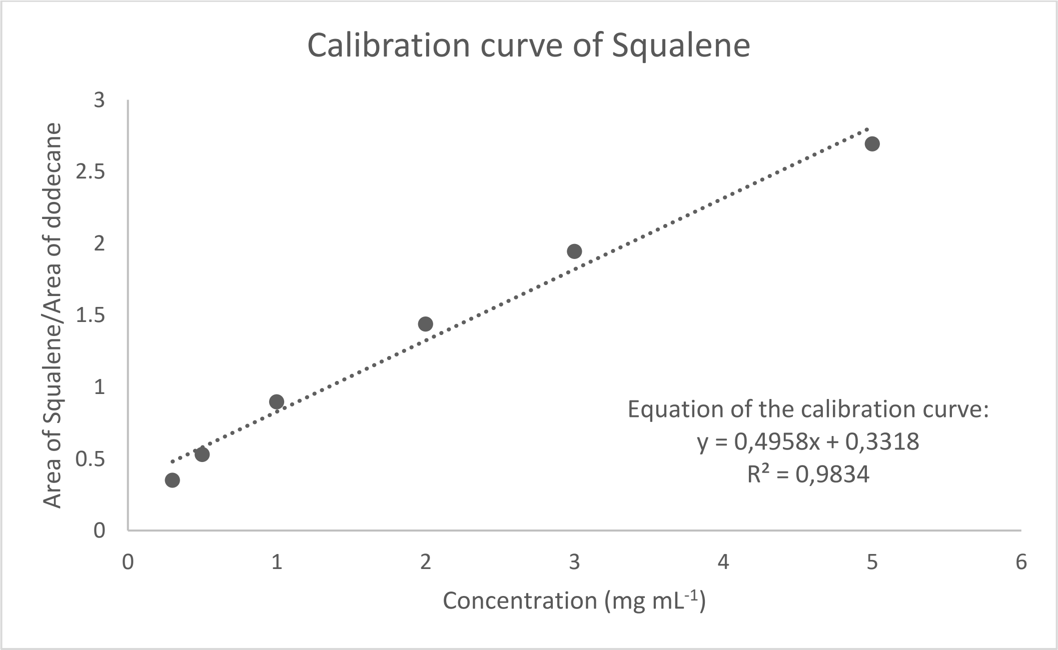

Figure S5. GC-MS calibration curve of Squalene

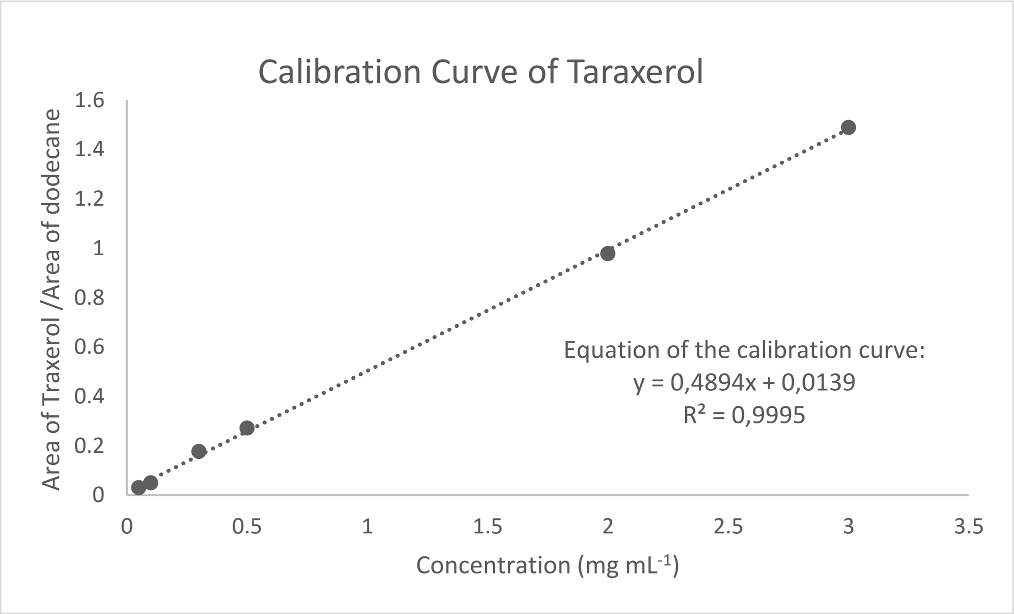

Figure S6. GC-MS calibration curve of Taraxerol

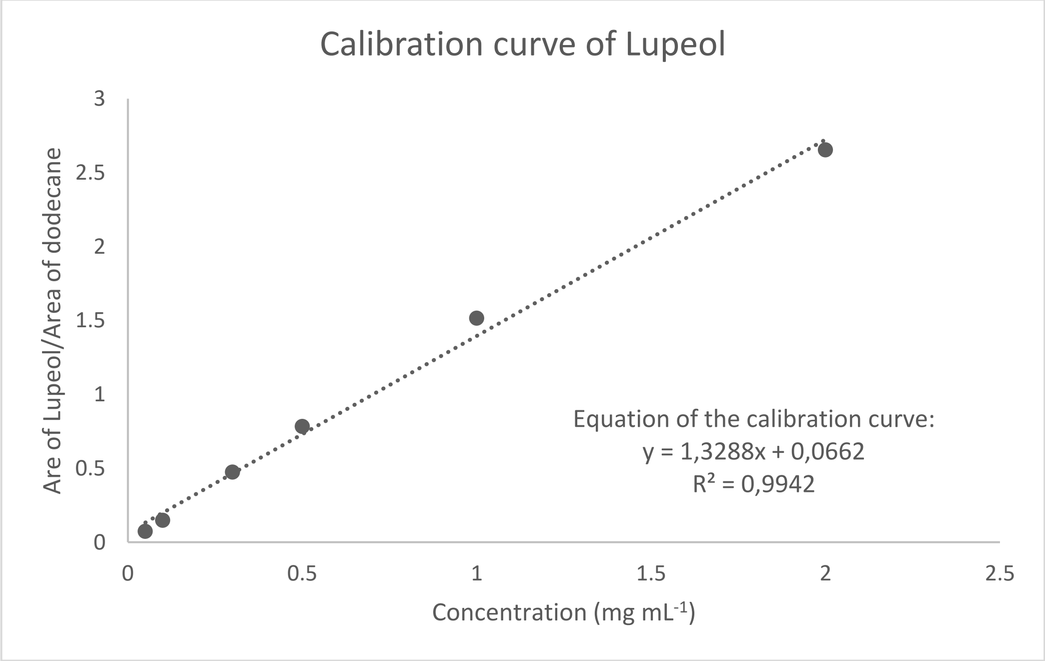

Figure S7. GC-MS calibration curve of Lupeol

References

1. Ramos, P.A.B., Guerra, Â.R., Guerreiro, O., Freire, C.S.R., Silva, A.M.S., Duarte, M.F., Silvestre, A.J.D. Lipophilic Extracts of Cynara cardunculus L. var. altilis (DC): A Source of Valuable Bioactive Terpenic Compounds. *J. Agric. Food Chem.* **2013**, 61, 8420–8429.
2. Palomino-Schätzlein, M., Escrig, P.V., Boira, H., Primo, J., Pineda-Lucena, A., Cabedo N. Evaluation of nonpolar metabolites in plant extracts by 13C NMR spectroscopy. *J Agric Food Chem.* **2011**, 59(21), 11407-16.
